# Cryo-soft X-ray tomography: using soft X-rays to explore the ultrastructure of whole cells

**DOI:** 10.1042/ETLS20170086

**Published:** 2018-03-29

**Authors:** Maria Harkiolaki, Michele C. Darrow, Matthew C. Spink, Ewelina Kosior, Kyle Dent, Elizabeth Duke

**Affiliations:** Diamond Light Source, Harwell Science and Innovation Campus, Didcot, Oxfordshire, U.K.

## Abstract

Cryo-soft X-ray tomography is an imaging technique that addresses the need for mesoscale imaging of cellular ultrastructure of relatively thick samples without the need for staining or chemical modification. It allows the imaging of cellular ultrastructure to a resolution of 25–40 nm and can be used in correlation with other imaging modalities, such as electron tomography and fluorescence microscopy, to further enhance the information content derived from biological samples. An overview of the technique, discussion of sample suitability and information about sample preparation, data collection and data analysis is presented here. Recent developments and future outlook are also discussed.

## Introduction

**Figure 1.**
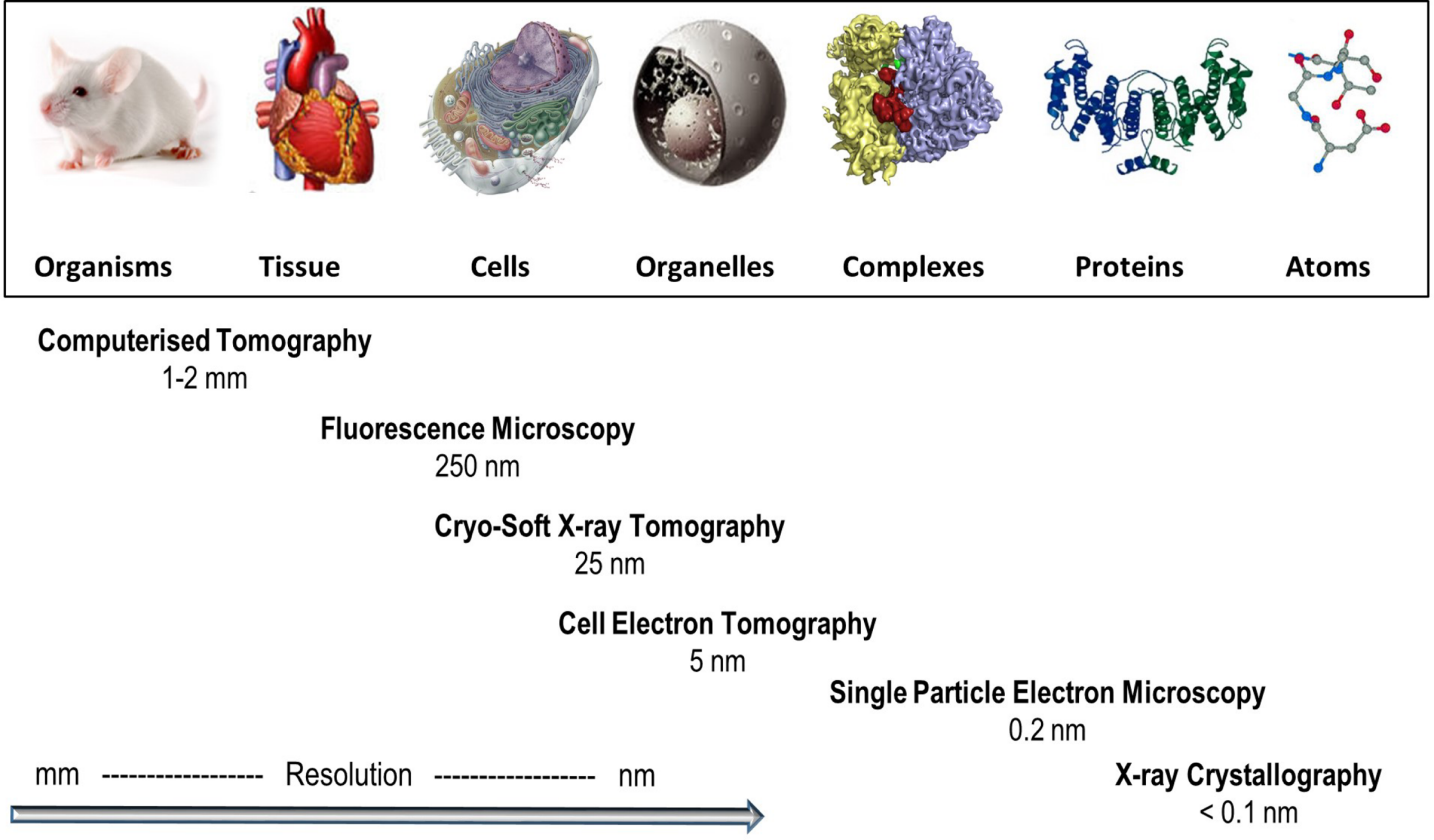
Biological imaging in context. Representative techniques developed for the investigation of structure in biological systems at different scales, in order of resolution attainable.

**Figure 2.**
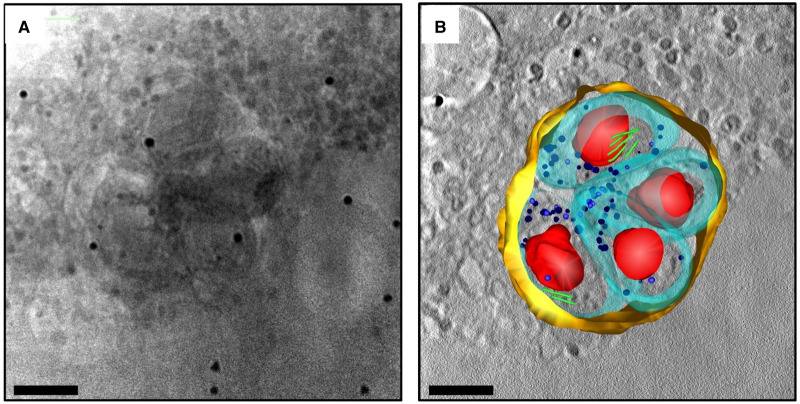
An example of typical cryo-SXT data. (**A**) A cryo-STX projection image of a *Toxoplasma gondii* containing vacuole within a human fibroblast cell. The reconstructed volume (**B**) shows the parasitophorous vacuole in yellow containing four parasites (plasma membranes in cyan and rhoptries in green) with their respective nuclei in red. The bars are 2.5 µm. Data collected at B24. Image courtesy of Katja Ota and Hellen Saibil (Birkberk College London, U.K.).

The imaging field in the life sciences has flourished in the past few decades and currently delivers 3D cellular imaging of impressive content at a range of resolutions (Figure 1). Electron tomography sets the bar in cellular imaging resolution [1,2], providing highly detailed views of thin biological samples, while fluorescence imaging, in conjunction with endogenous fluorescent labels, reveals intricate molecular interactions within whole cells [3,4], but at a lower resolution. Addressing this resolution gap, and as a direct result of a growing appetite for ever more diverse, cutting-edge imaging applications, cryo-soft X-ray tomography (cryo-SXT) has developed into a key player in the field. Cryo-SXT is a 3D imaging method for the visualisation of cellular ultrastructure at an intermediate resolution [5] and specifically addresses the need for detailed, 3D information on cellular features in thick specimens, such as whole cells, with little or no chemical or mechanical modification (Figure 2). It allows the observation of biological events on a range of scales, with imaging of details as fine as the internal structure of larger viruses and bacteria to the ultrastructure of yeast, protozoa and mammalian cells (Table 1A) and their associated components such as the nucleus, endoplasmic reticulum, mitochondria, parts of the cytoskeleton and more [47]. Many key cellular processes have been investigated to date with cryo-SXT such as chromatin rearrangement, virus–host interactions, cell motility, parasite life cycle, and lymphocyte activation and function (Table 1B).

**Table 1A:** Representative cells and organisms that have been examined by cryo-SXT

Bacteria	*Leptothrix orchacea* [6], *Escherichia coli* [7,8], *Mycobacterium smegmatis* [7], *Salmonella enterica* [9]
Yeast	*Saccharomyces cerevisiae* [10–12], *Saccharomyces pombe* [7,11–13], *Candida albicans* [7,11,12]
Algae	*Chlamydomonas reinhardtii* [14], *Scenedesmus*[15]
Plankton	*Emiliania huxleyi* [16]
Protozoa	*Plasmodium falcitatum* [17,18], *Trypanosoma brucei* [19], *Toxoplasma gondii* (Figure 3)
Viruses	Vaccinia [20,21], Herpes Simplex Virus 1 [22,23], Hepatitis C [24]
Primary cells	Erythrocytes [25,26], lymphocytes [9,12,22,27], olfactory sensory neurones [28,29]
Immortalised lines	EFN-R [30], HEK293 [31,32], G361 [33], RK13 [34], Cos7 [35], MCF-7 [36,37], Huh7 [38], RBL-2H3 [39], RAW264.7 [40], T-cell CEM [41], HT1080 [5], HT29 [15], J774 [42], PtK2 [20,43,44], 3T3 [42,45], DF1 [20], PC12 [19], mouse adenocarcinoma [25,46]

**Table 1:** B Representative biological processes studied with cryo-SXT to date

Virus–host interactions (membrane remodelling) [48]	Mitochondrial fission [35]
Chromatin reorganisation [22,23,28]	Phenotypic switching in yeast [45]
Assembly of viral factories [20]	Cholesterol crystal formation in macrophages [40]
*Plasmodium* haeme detoxification [17] and egress [49]	ER restructuring in HCV infection [24]
Clathrin-dependent cell motility in lymphocytes [41]	Calcium concentrates in algae [16]
Differentiation-induced chromatin distribution [28]	Nanoparticle adsorption by cells [42,50]
Erythrocyte physiology in disease [26]	Bacterial activation of T lymphocytes [9]
Degranulation in mast cells [39]	

Historically, the possibility of using X-rays to explore the ultrastructure of cells had been considered since the very discovery of X-rays by Röntgen in 1895 [51]. However, progress was hindered by the lack of X-ray sources and suitable optics until the 1970s when Fresnel zone plates first became available [52,53]. The following two decades saw intense effort concentrated on the development of X-ray microscopy for cellular imaging made possible by the availability of synchrotron sources, improved zone plates, specialised sample chambers and high-resolution direct detection cameras. By the 1990s, a thriving community of researchers was working hard to make use of the full capacity of the technique [54]. Pioneering in the area was the team of the Göttingen beamline at the Bessy storage ring in Berlin that went on to demonstrate the first use of X-ray microscopy on cryogenically preserved cells [43] (a sample preparation method previously validated in the field of electron microscopy [55,56]), thus heralding the dawn of cryogenic X-ray microscopy for biological specimens. Until this pivotal point, radiation damage from exposure to X-rays was a serious limitation in imaging biological samples [57]. Cryo-preservation significantly increased the radiation dose a sample could withstand prior to large-scale damage [58–60] and therefore allowed longer exposures and serial imaging, leading to the current implementations of cryo-SXT. Efforts since have led to the evolution of cryo-SXT into a user-friendly and accessible method [6,44,61] generating an ever-increasing volume of data (Table 1A,B). Further development of its capabilities and algorithm development for data processing, evaluation and analysis [10,19,62,63], and correlation with other imaging modalities [30,40,64,65] are currently areas of intense interest.

**Table 2:** Synchrotron-based soft X-ray tomography beamlines

	Beamline	Facility	Location
Operational	U41-TXM	Bessy II	Berlin, Germany
	XM2	Advanced Light Source (ALS)	Berkeley, California, U.S.A.
	Mistral	ALBA	Barcelona, Spain
	B24	Diamond Light Source (DLS)	Didcot, U.K.
Under construction	24A	Taiwan Photon Source	Hsinchu, Taiwan
	—	Shanghai Synchrotron Radiation Facility	Shanghai, China
	10ID1	Canadian Light Source	Saskatoon, Canada

**Figure 3.**
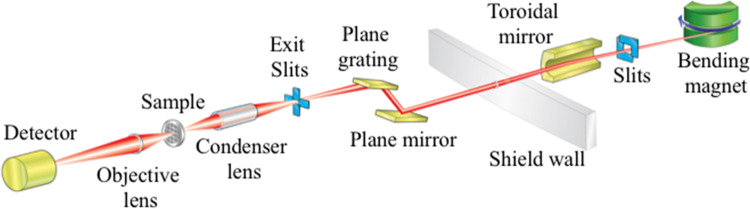
A schematic of beamline B24 at the Diamond Light Source synchrotron. X-rays are produced at a bending magnet, focused by a toroidal mirror and conditioned with a plane grating monochromator resulting in a secondary source at the exit slits. X-rays from his source are focused by capillary condenser lens and the resulting projections are directed via a zone plate objective to the detector.

Presently, cryo-SXT is available at many synchrotron beamlines across the globe [6,44,61] (Table 2) delivering high-contrast, cellular imaging up to a resolution of 25 nm [66]. For example, at the UK synchrotron, Diamond Light Source, the cryo-SXT beamline B24 (Figure 3 and Table 3) uses X-rays from a bending magnet on the synchrotron ring, which are passed to focusing and dispersive optics before reaching the microscope (UltraXRM-S220c Zeiss) vessel. There, they are delivered via a capillary condenser to the sample plane [67] and focused on the imaging plane of a camera with a Fresnel zone plate. Table-top set-ups using soft X-rays generated by a plasma source have also been described [68], but suffer from lower flux when compared with synchrotron sources, resulting in much longer acquisition times. Access to cryo-SXT is possible through many international synchrotron facilities (Table 1), where new users can work with technique experts to ascertain project feasibility and receive advice on experiment design.

**Table 3:** Beamline B24 design specifications

Beamline acceptance: 2 × 2 mrad
Beam focusing via a single toroidal mirror delivering 1 : 1 magnification
Plain grating monochromator with variable line spacing gratings delivering 200–2600 eV
Resolving power >1700
Beam size at sample: 20 µm
Photon flux at sample: 10^8^ photons/µm^2^
End Station: Xradia^*^ UltraXRM-S220c
Resolution: 25 and 40 nm (Xradia zone plate optic)
Detector: Direct detection CCD (Pixis-XO:1024B, Princeton Instruments)
Techniques possible: absorption- and phase-contrast modes
**Commissioned operation parameters**
Absorption contrast at 500 eV (water window) with a 40 nm zone plate
Samples mounted or grown on 3 mm grids, 4× grids loaded at a time
Magnification delivered:
Data collection strategy: tilt series −70° to +70°, 0.5° steps
Data handling: automated backup and reconstruction
In-line fluorescence detection capability

* Xradia is now part of the Carl Zeiss X-ray Microscopy group.

## Theory of cryo-SXT

In cryo-SXT, 2D images are formed when an X-ray beam travels through an object and is differentially attenuated or scattered by structures in its path [54]. When X-rays emerge from the sample, they effectively carry the likeness of that object, which they deliver to a camera, resulting in a 2D projection of the 3D sample. Single 2D projections, however, provide only limited volumetric information since they carry no information about the relative depth of various features. To generate a 3D volume of the contents of a cell, 2D projections are recorded at discrete angular increments about a sagittal rotation axis to create a tilt series of images that present structural features at different relative orientations [1]. Tilt series are then reconstructed into tomograms, via either Fourier or real-space methods, to produce a volumetric representation of cellular contents [69]. These tomograms are effectively the cellular equivalent of the common computed tomography (CT) scans routinely performed in medical settings.

Cryo-SXT can deliver images either by absorption contrast at defined spectral areas [54] or by phase contrast through a reference wave front at higher X-ray energies [70]. Absorption contrast is the current standard in the field and it takes advantage of the absorption characteristics of biological matter at the ‘water window’ [the spectral region defined by the X-ray absorption edges of carbon (284 eV) and oxygen (543 eV)] [71]. X-rays within this region are absorbed an order of magnitude more by carbon- and nitrogen-rich biological structures when compared with the oxygen-rich medium that surrounds them. This relative difference generates contrast in the projections of a cellular area.

There are many inherent benefits in cryo-soft X-ray imaging. Given the natural absorption contrast resulting from carbon-rich biological structures, there is no need for additional sample staining. Moreover, the penetration depth of soft X-rays through biological matter is several microns, and as a result, X-rays can effectively travel the width of a single cell without substantial dissipation; cells therefore do not need to be sectioned or milled to suitably thin samples [54]. Finally, due to cryo-preservation, chemical fixation (to minimise sample drift during data collection and confer resistance to radiation damage) is unnecessary [43]. Hence, cryo-SXT can deliver structural information on cellular ultrastructure on whole cells in a near-native state. Correlation with further imaging modalities is another appealing feature of cryo-SXT. The 3D volume displaying the ultrastructure of a cell produced by cryo-SXT can be correlated with 2D and 3D fluorescence microscopy to localise events or features of interest. This allows for the evaluation of molecular function with respect to localisation within a cell. Many studies, to date, have used fluorescence microscopy and cryo-SXT correlative approaches, showcasing the wealth of detailed information that can be acquired in this way [13,24,25,27,30,31,34–36,39,40,45,64].

In summary, cryo-SXT is an imaging modality that can provide ultrastructural information, with a resolution range of 40–25 nm, of a whole, intact cell, without the need for fixation, dehydration or staining to prepare the sample for imaging. It has generally been used to study whole cells; however, imaging tissue preparations and non-biological composites is also possible with cryo-SXT. Using this technique, especially in conjunction with other correlative imaging modalities, questions about the effects of stimuli or treatment, or the process of disease or infection, can be studied.

## Cryo-SXT workflow

**Figure 4.**
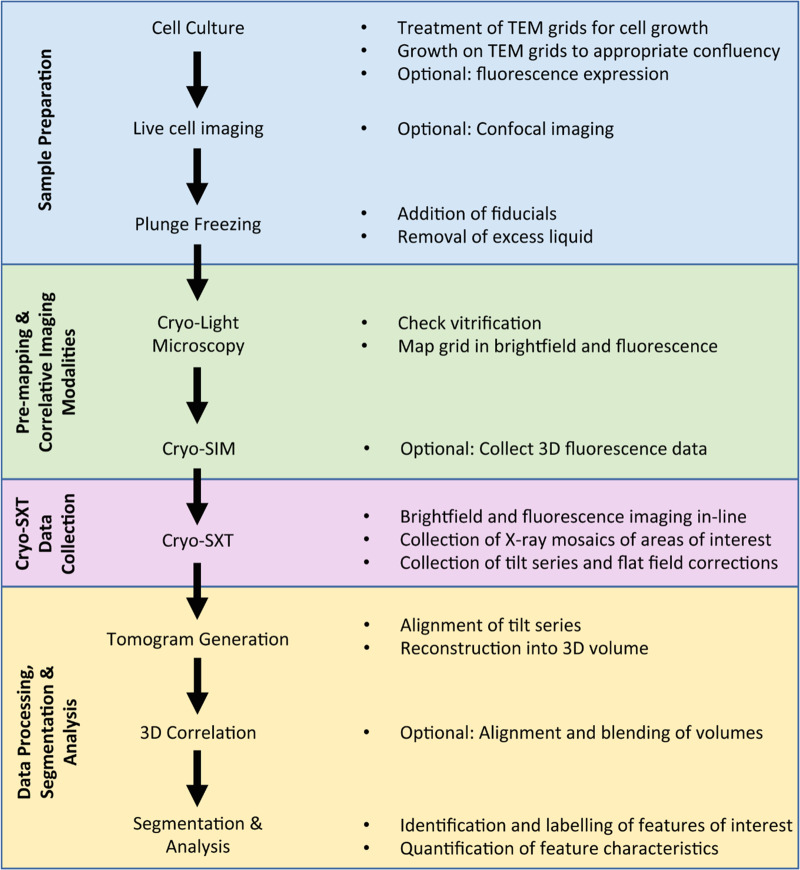
Cryo-SXT workflow.

Here, we provide an example workflow describing the steps involved in preparing samples for cryo-SXT, followed by acquiring and processing cryo-SXT data (Figure 4).

### Sample preparation

For the purposes of this review, the discussion will focus on experimental parameters that are relevant to beamlines, such as B24, that are designed to use standard or modified electron microscopy grids [44,64]. An alternative set-up is used at the XM2 beamline at the ALS synchrotron where samples in suspension can be loaded into thin-walled glass capillary tubes [61] that serve as sample holders.

Typical cryo-SXT samples are adherent or non-adherent cells; however, the technique can also accommodate the imaging of tissue sections or separated cellular contents. Often, fluorescent markers are endogenously expressed or added to cell culture media. For adherent cell lines, the cells are cultured on 3.05 mm gold transmission electron microscopy (TEM) grids [72]. Prior to cell culture, the grids are coated with a carbon substrate [30,31] to provide a retaining surface for cells and that surface is further treated by either glow discharge or plasma cleaning to increase hydrophilicity. To encourage cell attachment and spreading, the carbon film may also be treated with adherence-promoting agents such as ornithine, poly-d-lysine or laminin [73–75]. Conventional brightfield microscopy can be used to evaluate cell attachment and growth on the grids with the general goal of ∼40% confluency and a good distribution of individual cells. Fluorescence detection techniques, such as confocal microscopy, can be used to document successful endogenous expression of fluorescent markers.

Plunge freezing [76] is currently the method of choice for sample preservation for cryo-SXT, although high-pressure freezing [77] is better suited to thicker samples (>10 µm). In plunge freezing, vitrification is achieved via rapid cooling to temperatures below −170°C by plunging the sample grid into liquid nitrogen-cooled liquid ethane or propane. Prior to this step, gold nanoparticle fiducial markers are added (100–250 nm diameter) for later use during the process of tilt series reconstruction [31] and excess liquid is blotted away reducing the depth of the sample, ideally to just thicker than a single layer of cells. After plunge freezing, samples can be stored at liquid nitrogen temperatures indefinitely.

### Pre-mapping and correlative imaging

**Figure 5.**
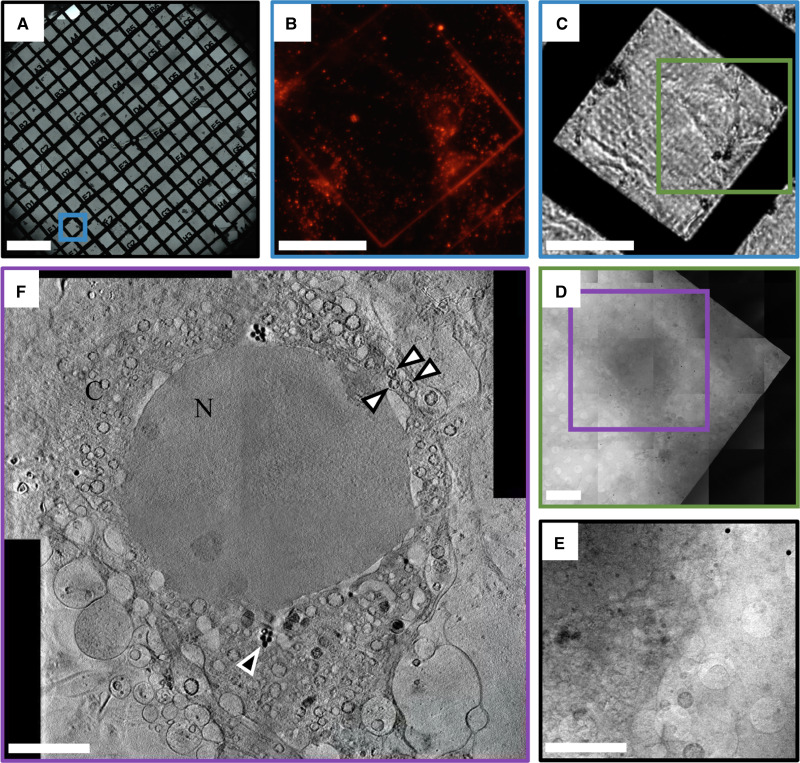
Typical cryo-STX imaging progression for adherent cells with fluorescence labels. (**A**) Brightfield grid overview of cryo-preserved mouse primary neuronal cells on a TEM grid (bar = 500 µm), (**B**) close-up fluorescence and (**C**) brightfield images of the area on **A** marked by a blue square (bar = 50 µm), (**D**) X-ray mosaic of the area marked in **C** (bar = 10 µm), (**E**) single field or view X-ray projection and (**F**) slice through a cumulative tomogram, showing the nucleus (N) and surrounding vesicles in the cytoplasm (C) produced by stitching four tomograms collected in adjacent overlapping areas. Bars for **E** and **F** = 5 µm; black arrow with white outline points to lipid droplets, white arrows with black outline point to mitochondria; Data collected at B24. Image courtesy of Karen Marshall and Louise Serpell at Sussex University, U.K.

Samples should be evaluated for suitability prior to a cryo-SXT experiment, as plunge freezing can be unsuccessful for many reasons. A cryostage-equipped light microscope can be used to examine grids and confirm that cells have retained their gross morphology, and that the grid does not have ice contamination or many large breaks in the support film. Samples can also be pre-mapped in brightfield and fluorescence (Figure 5A,B), as appropriate, and areas of interest can be identified for 3D data collection. If fluorescent markers are present, it is entirely appropriate at this point to examine their distribution with super-resolution fluorescence cryo-microscopy methods before the fluorophores present are compromised by exposure to X-rays. 3D super-resolution imaging techniques, such as cryo-structured illumination microscopy (cryo-SIM) [78] (available on beamline B24 through a collaboration with the advanced bioimaging unit, Micron, at the University of Oxford, UK), are particulary suited to correlation with cryo-SXT as they can provide a stack of relatively high-resolution images (∼200 nm) through the sample, mapping in 3D the intracellular location of fluorophores.

### Cryo-SXT data collection

**Figure 6.**
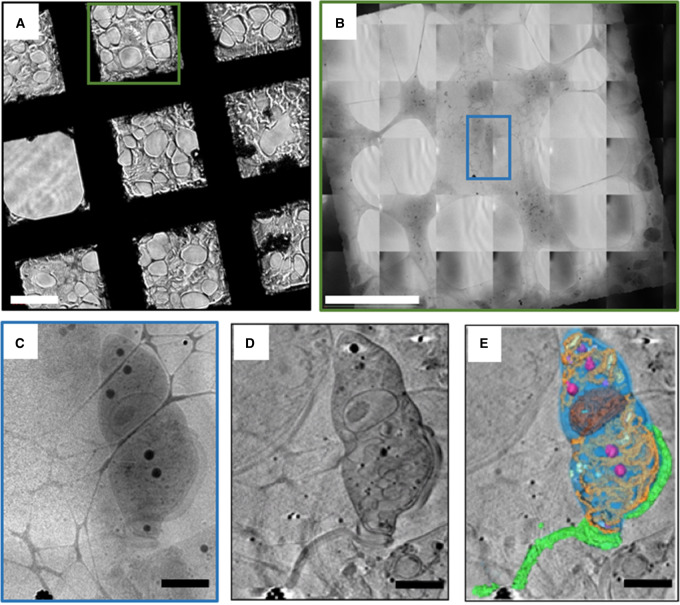
Typical cryo-STX imaging workflow for smaller samples (*Trypanosome brucei*) with no associated fluorescence. (**A**) Brightfield overview of cryo-preserved cells on an area of holey carbon on a TEM grid (bar = 50 µm), (**B**) X-ray mosaic of the area on **A** marked by a green square (bar = 25 µm), (**C**) X-ray projection of the area on **B** marked by a blue square, (**D**) tomogram slice of the same area after reconstruction and (**E**) segmented volumes with delineated cellular ultrastructure (flagellum is green, cell body in blue and organelles in various other colours). Data collected at B24. Image reproduced from Luengo et al. [19].

At beamline B24, sample grids are placed into the X-ray microscope vessel using a transfer chamber that allows the transition from liquid nitrogen storage under atmospheric pressure to cooling via conduction under high vacuum. Grids are placed at the imaging position in close proximity to the microscope optics (during imaging, optics are only few millimetres away) where they can be examined using an in-line conventional light microscope (20× objective at B24) for a final inspection of the sample and the opportunity to locate the exact areas previously mapped at higher resolutions in other imaging modalities. Because the light microscope is in-line, a common set of working co-ordinates can be assigned in order to return to the same area for X-ray imaging (Figures 5C and 6A).

Whole grid squares are examined using low-dose X-ray exposures and collation of adjacent stills into mosaic images (Figures 5D and 6B), which provide overviews of whole cells in the region of interest. Mosaics are needed because the field of view of transmission X-ray microscopes tends to be 20 µm or less and adherent cells are often substantially larger. Specific areas are identified for tilt series acquisition and for each, the maximum rotation prior to obstruction and appropriate angular steps and exposure times are determined. If a single field of view is insufficient for the experiment, adjacent overlapping tilt series can be collected and later computationally merged (Figure 5F). Generally, for a flat TEM grid, tilt series are collected from ±65° with a step size of 0.5° or 1°. Exposure times are dependent on X-ray flux and sample thickness, but are generally in the range of a few seconds per projection. Depending on the parameters chosen, tilt series acquisition generally takes ∼5–20 min in total. Background flat field images are collected regularly to correct for fixed pattern noise present in the beam.

For a flat TEM grid, the maximum rotation possible is limited both by the attenuation of the beam due to increased sample thickness at high tilts and by the physical proximity of the X-ray optics to the grid. This limited rotation tilt series results in ‘missing wedge’ artefacts, named after the shape of the missing information in Fourier space. The rotation restriction is less problematic when using a modified, flat grid [64] and in the case of a glass capillary tube, a full rotation tilt series can be collected, alleviating the issues created by the missing wedge [6]. However, currently, glass capillary tubes are more suited to imaging cell suspensions, while flat grids can accommodate both cell suspensions and adherent cell preparations. Irrespective of the sample holder, the depth of focus at the sample plane is generally smaller than the total depth of the sample being imaged, leading to some blurring of out-of-focus features and possible loss of resolution during the reconstruction process [15].

More sophisticated data collection techniques in cryo-SXT, such as dual tilt series to reduce missing wedge artefacts [80] and focal series data acquisition to circumvent the resolution reduction due to out-of-focus information [15]), are currently pioneered by the Mistral team at ALBA [15] and are likely to be adopted elsewhere soon.

### Data processing, segmentation and analysis

At synchrotrons, as data are collected, they enter a semi- or fully-automated processing pipeline that handles changes in file format, alignment of the tilt series and finally reconstruction into a 3D tomogram. This allows for an initial near real-time data analysis, enabling evaluation of data quality during the data collection process. At B24, for example, tilt series are aligned and reconstructed using the batch reconstruction processes in IMOD [81]. Other in-house alignment and reconstruction software, which is specific to X-ray imaging, is also used elsewhere [63].

The generation of a tomogram from a tilt series depends heavily on the accurate alignment of images within that series. During data collection, individual images can suffer from mechanical misalignments such as rotation drift or motor backlash. If these misalignments are not corrected, they can result in loss of resolution, or the blurring of features, in the 3D tomogram. Fiducial markers, either added during sample preparation or natural fiducials found in the sample, are generally used to align the tilt series. However, it is also possible to align tilt series using patch-based cross-correlation algorithms [79]. In any case, the features of the fiducials, or within the patches, must be high contrast and unique so that their movement can be accurately tracked.

Generally speaking, two main reconstruction algorithms are used in the field of cryo-SXT: weighted back projection (WBP) [82] and simultaneous iterative reconstruction technique (SIRT) [83]. WBP is a Fourier space reconstruction method which can accentuate high-resolution noise, while SIRT is performed in real space, and depending on the number of iterations chosen, can filter high-resolution noise from the resulting tomogram.

While many subcellular organelles can be identified in cryo-SXT tomograms, segmentation is necessary to both meaningfully visualise and analyse this data (Figure 5). Segmentation is the process of delineating and classifying objects or areas of interest throughout the 3D volume. This process can be entirely manual, with a person outlining the boundaries of cellular structures of interest on each slice through the 3D volume [81,84]; or segmentation algorithms can be used to decrease the time and manual input required [19,85–87]. Manual segmentation can be, by far, the most time-consuming step in a cryo-SXT experiment and it is generally regarded as somewhat objective and variable [88]. Recent advances in data representation and segmentation have been introduced at Diamond Light Source through the SuRVoS Workbench, which uses minimal manual training inputs to assign voxels in the volume to user-defined classes [19,89]. Once cellular features have been segmented and classified, further analysis of size, shape and localisation within the context of a whole cell can lead to biological understanding.

Data processing and analysis in the cryo-SXT field is currently in its formative years. While many approaches and software packages can be borrowed and adapted from other fields, ongoing efforts are underway to provide purpose-built software packages specific to the needs of the cryo-SXT community.

## Conclusion and future direction

Currently, cryo-SXT offers an excellent way to visualise the ultrastructure of cells, in order to delineate organelle architecture, membrane organisation and interactions within the cell. In addition, the use of cryo-SXT as a correlative imaging technique can provide large-scale cellular context for data collected via electron tomography or fluorescence microscopy.

Cryo-SXT is still an emerging technique for the life sciences, with a growing user community that will inevitably direct its development and nurture it to maturity. Technical implementation, sample protocols and related applications are widely documented, although the technique does, by and large, remain within the realm of large infrastructures such as synchrotrons. Access through synchrotron sources, however, is available to a great number of researchers across the world and as more cryo-SXT beamlines emerge from commissioning phases, better coverage is projected.

In the future, it is expected that cryo-SXT will deliver better contrast at higher resolutions; data collection, processing and segmentation pipelines will improve, ideally to the point of full automation; associated developments in correlative imaging will ease the current physical and software difficulties in transitioning from one technique to another and cryo-SXT will be established as a robust partner in interdisciplinary approaches.

## Summary

- Cryo-SXT offers near-native imaging of whole cells.
- The method uses the natural absorption of soft X-rays by biological matter to produce projection images of cells.
- Cryo-SXT data can be correlated to fluorescence microscopy and electron microscopy/tomography data.
- Access to the technique is provided primarily via synchrotron facilities.
- Sample preparation methods are well defined and documented.
- Cryo-SXT data handling and analysis protocols are under development.

## Abbreviations

cryo-SXT: Cryo-soft X-ray tomography
SIRT: simultaneous iterative reconstruction technique
TEM: transmission electron microscopy
WBP: weighted back projection
